# Unveiling the frontiers of potato disease research through bibliometric analysis

**DOI:** 10.3389/fmicb.2024.1430066

**Published:** 2024-07-03

**Authors:** Ling Weng, Zhurui Tang, Muhammad Fahad Sardar, Ying Yu, Keyu Ai, Shurui Liang, Jawaher Alkahtani, Dianqiu Lyv

**Affiliations:** ^1^College of Agronomy and Biotechnology, Southwest University, Chongqing, China; ^2^Chongqing Key Laboratory of Biology and Genetic Breeding for Tuber and Root Crops, Chongqing, China; ^3^Key Laboratory of Germplasm Innovation of Upper Yangtze River, Ministry of Agriculture and Rural Affairs, Chongqing, China; ^4^Engineering Research Center of South Upland Agriculture, Ministry of Education, Chongqing, China; ^5^Key Laboratory of Ecological Prewarning, Protection and Restoration of Bohai Sea, Ministry of Natural Resources, School of Life Sciences, Shandong University, Qingdao, China; ^6^Soil and Fertilizer Institute, Anhui Academy of Agricultural Sciences (National Agricultural Experimental Station for Soil Quality, Taihe)/Key Laboratory of Nutrient Cycling and Arable Land Conservation of Anhui Province, Hefei, China; ^7^Department of Botany and Microbiology, College of Science, King Saud University, Riyadh, Saudi Arabia

**Keywords:** potato, disease, control strategies, bibliometrics, biocontrol

## Abstract

Research on potato diseases had been widely reported, but a systematic review of potato diseases was lacking. Here, bibliometrics was used to systematically analyze the progress of potato disease. The publications related to “potato” and “disease” were searched in the Web of Science (WOS) from 2014 to 2023. The results showed that a total of 2095 publications on potato diseases were retrieved, with the annual publication output increasing year by year at a growth rate of 8.52%. The main countries where publications were issued were the United States, China, and India. There was relatively close cooperation observed between China, the United States, and the United Kingdom in terms of international collaboration, while international cooperation by India was less extensive. Based on citation analysis and trending topics, potential future research directions include nanoparticles, which provides highly effective carriers for biologically active substances due to their small dimensions, extensive surface area, and numerous binding sites; machine learning, which facilitates rapid identification of relevant targets in extensive datasets, thereby accelerating the process of disease diagnosis and fungicide innovation; and synthetic communities composed of various functional microorganisms, which demonstrate more stable effects in disease prevention and control.

## 1 Introduction

Potatoes (*Solanum tuberosum* L.) were the fourth-largest food crop, playing a vital role in ensuring global food security. However, susceptibility to plant pathogenic bacteria, pathogenic fungi, and viruses during cultivation poses significant challenges, adversely affecting both the yield and quality of potatoes (Birch et al., 2012). Currently, potato diseases and pests can be classified into fungal, bacterial, viral, and insect categories based on their respective pathogens. Fungal diseases include late blight (*Phytophthora infestans*), black scurf (*Rhizoctonia solani*), and black dot (*Colletotrichum coccodes*) (Smirnov, 2003; van de Vossenberg et al., 2022; Sanzo-Miro et al., 2023). Bacterial infections include common scab (*Streptomyces* spp.) and bacterial wilt (*Pectobacterium* spp.), among others (Majeed and Muhammad, 2020; Tessema and Seid, 2023). Virus-related ailments mainly consist of mosaic virus, Y virus, and tobacco rattle virus (Moyo et al., 2022; Dupuis et al., 2023), with viroids emerging as a significant concern affecting potatoes (Flores et al., 2020). Pests primarily include nematodes, psyllids, whiteflies, which not only directly impact the quality of potato tubers but also facilitate the spread of pathogens, making potatoes more vulnerable to disease (Suffert and Ward, 2014; Gamarra et al., 2020; Prager et al., 2022; Dupuis et al., 2023). Overall, the quality and yield of potato tubers suffer significantly from the infestation of various pathogens, necessitating timely disease prevention and control measures to mitigate substantial economic losses.

Reasonable disease control strategies effectively suppressed the level of potato diseases. Current disease control strategies included field management, breeding resistant varieties, chemical pesticides, and biocontrol. Field management involved appropriate cultivation practices to reduce the number of pathogens and achieve disease control. For example, after continuous cropping of potatoes, harmful pathogens accumulated in the soil over the years, exacerbating the occurrence of diseases. Practices such as crop rotation and intercropping altered the structure of soil microbial communities, thereby reducing pathogen abundance to ensure potato productivity (Li et al., 2023). Chemical control involved applying chemical agents to kill pathogens and pests to achieve control. For example, in controlling bacterial diseases of potatoes, fungicides such as fluazinam, 2,4-dichlorophenoxyacetic acid benzothiazole, and mancozeb were applied in forms such as seed coating, sprays, and soil additives (Hosny et al., 2014; Yuliar and Toyota, 2015). Chemical control was the mainstream method in production, but excessive use of chemical agents could lead to pathogen resistance and serious soil and environmental pollution (Lin et al., 2021; Nadgir and Biswas, 2023). Biocontrol utilized various beneficial organisms and their bioactive substances to control disease and pest proliferation, aiming to suppress or even eliminate disease and pest damage. Common beneficial microorganisms included *Bacillus, Pseudomonas, Trichoderma*, and *Streptomyces*, which reduced the severity of diseases by secreting active antibacterial substances (Vejan et al., 2016). However, among the numerous control strategies, there was a lack of systematic review of potato diseases, including the types of diseases in major countries, current mainstream control measures, and future research directions, which would contribute to the advancement and rapid application of potato disease control technologies.

This problem can be solved with bibliometrics analysis, a discipline based on Bradford's, Lotka's, and Chipf's laws, as well as other empirical distribution laws. Through citation analysis, keyword analysis, and source analysis, the evolution of potato pests and diseases can be quantitatively described, as well as the current research hotspots and future research directions (Zupic and Cater, 2015). One of the key advantages of bibliometrics lies in its lack of geographical constraints, allowing for data collection and analysis across specific regions or timeframes. Moreover, specialized analysis software can process bibliometric results and present them in a visually accessible format. Presently, bibliometric analysis finds application across various disciplines, including medicine, chemistry, energy, computer science (Ariza-Guerrero and Blazquez, 2023; Baako and Abroampa, 2023; Huang et al., 2023; Tang et al., 2023; Xu et al., 2023).

In this study, we propose to use bibliometric to analyze the leading countries, authors, and author keywords in terms of the number of publications, with the aim of obtaining the current status of research on potato diseases, including the main types of diseases studied in each country, the main prevention and control ideas for the diseases, and the possible directions of research in the future.

## 2 Materials and methods

In this study, we conducted a bibliometric analysis of the literature published in the field of potato diseases from 2014 to 2023, choosing the Web of Science core collection as the data source. Because “disease” is not always used as a keyword in some articles on potato diseases, we used “potato” and “disease” as the search terms for keywords and abstracts, and the date range was set to 2014–2023.

In addition, searches for “potato” and “disease” also included literature related to potato and human diseases, such as the effects of potato and human intestinal diseases, so irrelevant literature was manually eliminated to form the analyzed literature set. Data analysis and visualization were performed using the bibliometrix, ggplot, visNetwork, and igraph packages in R_v4.3.2 and Cytospace_v3.10.1 (Aria and Cuccurullo, 2017).

## 3 Results

### 3.1 Overview of publications

**Figure 1:**
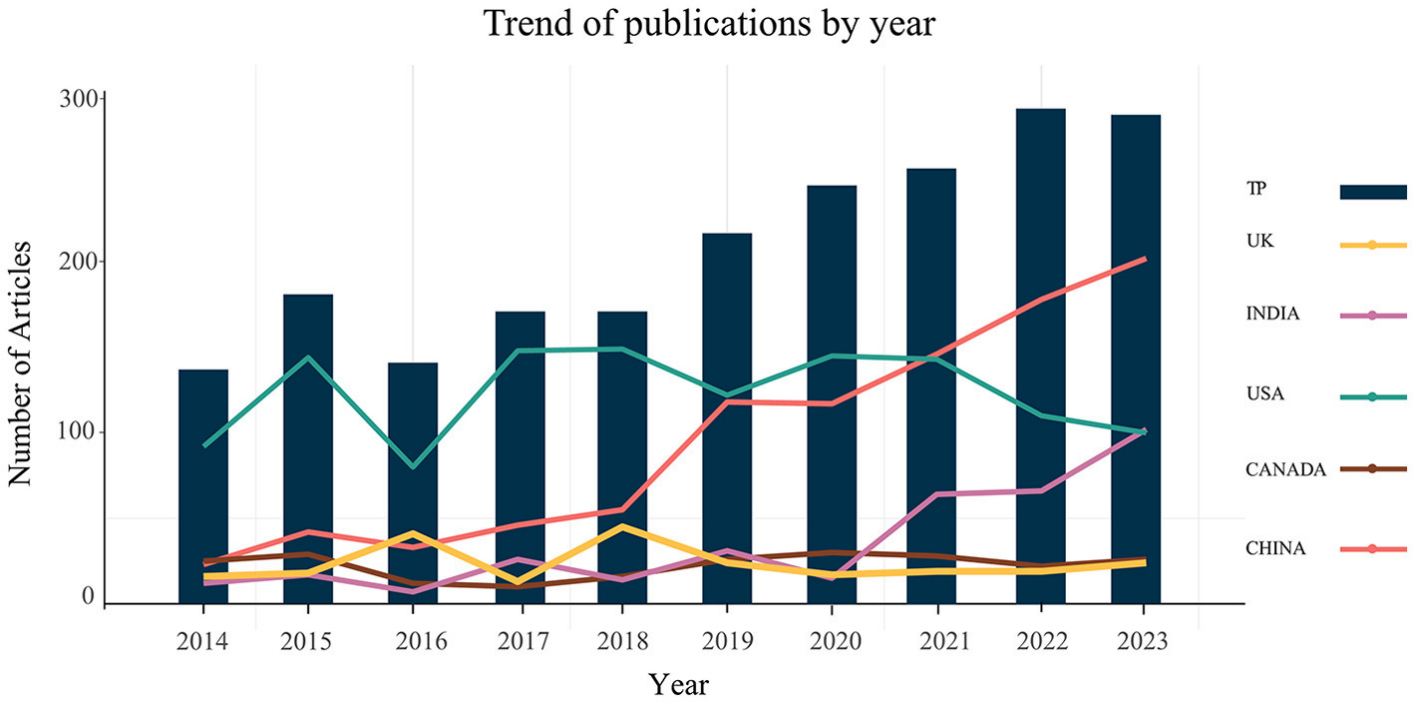
Trends in the number of published articles related to potato disease by year. TP, total papers.

During the period from 2014 to 2023, a total of 2,095 papers focusing on potato pests and diseases were published by authors from 103 countries. This corpus comprised 1,923 articles and 111 review articles. These publications were spanned across seven languages, with the majority, 2057 (98.19%), being in English, followed by 19 (0.91%) in Spanish, 7 (0.33%) in Chinese, 7 (0.33%) in German, 3 (0.14%) in Turkish, 1 (0.05%) in Polish, and 1 (0.05%) in Russian. The analysis of the total number of articles revealed an average annual growth rate of 8.52%, with a notable surge observed in the number of articles published between 2018 and 2022 (Figure 1). This result suggests that, with global food supply pressures increasing dramatically, ensuring potato yield and quality by mitigating pests and diseases had become the consensus of most people.

### 3.2 Analysis of the issuance of documents and international cooperation by major countries

The United States led in the number of publications within the field of potato diseases, followed by China and India, comprising 23.01, 18.57, and 7.21% of the total publications, respectively. Despite shifts in the ranking of total citations, the United States remained at the forefront, followed by China, with the Netherlands securing third place (Table 1). It was also found that India was well-ahead of the UK, Canada, and New Zealand in terms of annual publications in 2021, but it was still far behind the US and China (Figure 1). After 2018, the gap in total publications between China and the United States continued to narrow. This could be attributed to the national policy support and increased research funding following the Chinese government's proposal of the strategy to promote potatoes as a staple food in 2016 (Ministry of Agriculture Rural Affairs of the People's Republic of China, 2016). Among the top 20 countries, seven were located in Europe, seven in Asia, four in the Americas, and one each in Australia and Africa, underscoring the global prominence of potato pests and diseases.

**Table 1:** Country ranking of the number of papers published.

**Country**	**Frequency**	**TC**	**ACPP**	**TNCC**
USA	1,233	5,903	16.30	207
China	960	3,992	11.80	191
India	354	1,036	7.80	22
Canada	224	1,349	14.20	53
Netherlands	183	2,291	43.20	93
Egypt	184	413	8.60	39
Germany	154	643	14.60	38
UK	236	2,099	33.90	131
Poland	152	784	13.50	27
Australia	133	643	12.90	37
Pakistan	153	273	8.00	47
France	115	326	11.20	40
Sweden	111	644	16.90	35
Japan	123	414	9.60	13
Peru	88	117	6.2	58
Iran	95	362	8.60	14
Russia	111	299	6.80	3
Israel	83	206	9.4	28
South Korea	116	366	11.10	20
Brazil	94	417	12.30	16

TC, total citations; ACPP, average citations per publication; TNCC, Total numbers of cooperation between countries.

**Figure 2:**
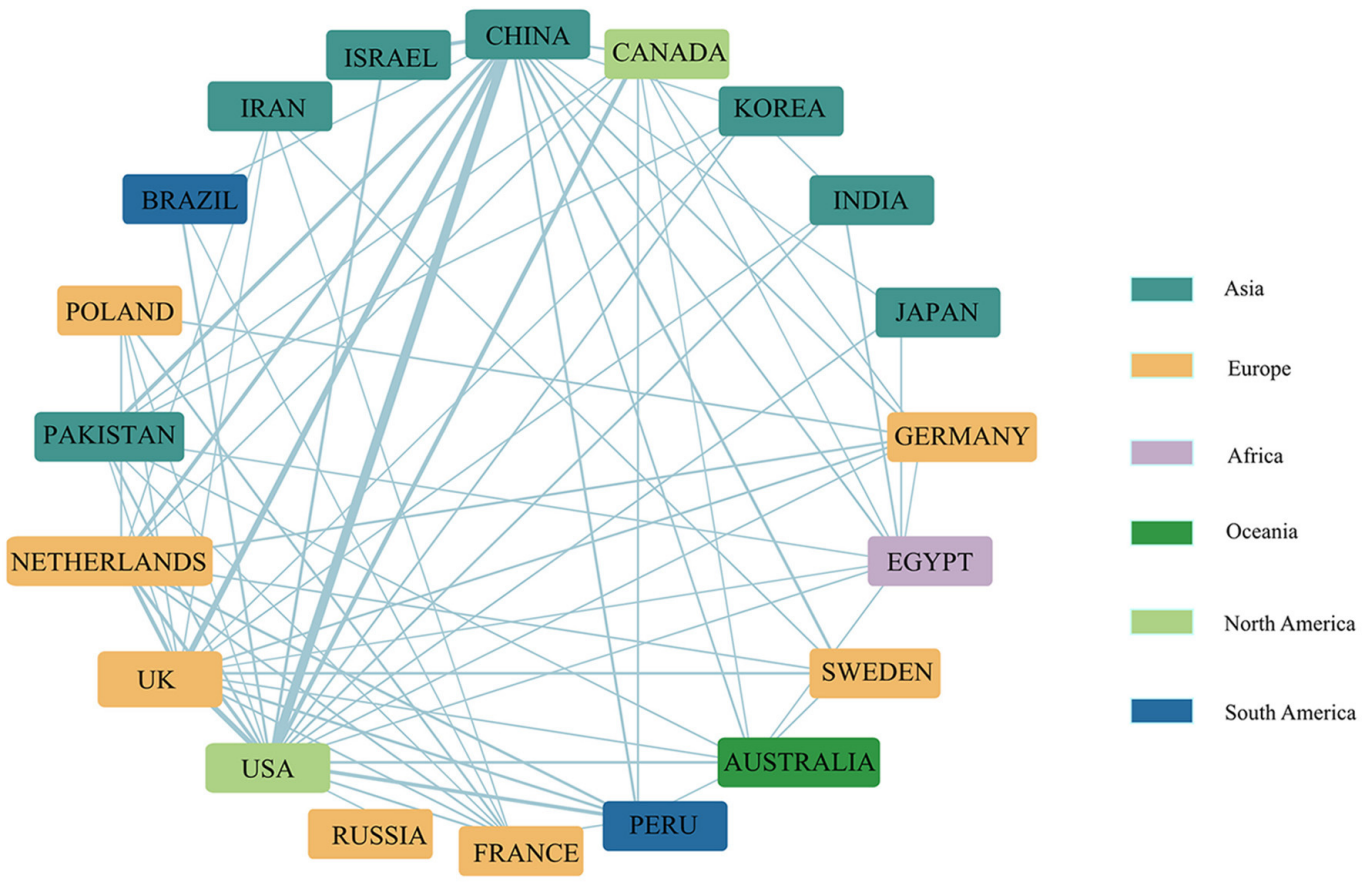
The top 20 national cooperation networks with the highest number of papers. The connection thickness represents the number of cooperation between countries, and the node size reflects the overall cooperation frequency of the country.

International cooperation enhanced the impact of the discipline by facilitating the flow and integration of knowledge, as different countries and regions had different focuses in potato disease research. Cooperation led to the transfer of technology, inspired innovation, and ultimately fulfilled the scientific mission (Borodiyenko et al., 2023). Total numbers of cooperation between countries represented the total number of times the country has cooperated with other countries. The United States, China, the United Kingdom, and Germany were found to have better cooperation with other countries and to be more focused on international cooperation (Table 1). The highest frequency of cooperation was between China, the United States, and the United Kingdom, followed by cooperation between Canada and United States, was second only to the cooperation frequency between China, the United States (53) and China and Britain (30), while international cooperation by India was less extensive (Figure 2).

### 3.3 Contribution of leading authors

Statistics related to authors provided insight into the top experts in the field of potato diseases, and research by these experts shed light on the cutting-edge development direction of potato diseases. Out of 2095 articles, 7,814 authors were accounted for, including 5,942 authors who contributed only one article, 386 authors who contributed three articles, and 58 authors who contributed ten or more articles. The top 20 authors in terms of the number of articles published had contributed 394 articles, representing 17.86% of the total number of articles published (Table 2).

**Table 2:** Contributions from the top 20 authors in the field of potato diseases.

**Author**	**TP**	**TC**	**h_index**	**g_index**	**m_index**	**ACCP**	**Country**
Munyaneza JE	19	449	15	19	1.364	24	USA
Sharma S	34	402	13	19	1.182	12	India
Andreasson E	21	387	12	19	1.091	18	Sweden
Karasev AV	20	298	12	17	1.091	15	USA
Birch PRJ	15	842	12	15	1.091	56	Birch
Cooper WR	20	340	11	18	1.000	17	USA
Liljeroth E	19	391	11	19	1.000	21	Eeland
Gudmestad NC	20	234	10	14	0.909	12	USA
Rush CM	19	236	10	15	0.909	12	USA
Visser RGF	16	381	10	16	0.909	24	Netherlands
Lojkowska E	15	324	10	15	0.909	22	Poland
Rashed A	17	194	9	13	0.818	11	USA
Tamborindeguyc	20	236	8	15	0.727	12	USA
Wilson CR	19	187	8	13	0.727	10	Australia
Tegg RS	18	183	8	13	0.727	10	Australia
Kumar R	21	161	7	12	1.000	8	India
chakrabarti SK	16	118	7	10	0.636	7	India
GEVENS AJ	15	406	7	15	0.636	27	USA
Johnson DA	15	300	7	15	0.636	20	USA
Kumar A	15	72	4	8	0.400	5	India

TP, total papers; TC, total citations; ACPP, average citations per publication.

The most articles in the field were published by Sharma, who ranked second in the h-index, with the number of publications and the average annual number of citations by this author increasing year by year over the selected time period (Supplementary Figure S1). It was shown that the influence of this author was gradually increasing. Differentially expressed genes in nematodes, *pathogenic oomycetes*, and *fusarium sporotrichum* infested potatoes were screened for by this author, with the aim of finding resistance genes and breeding highly resistant varieties. Additionally, tools for automated and rapid pest and disease detection based on machine learning, image recognition, and other techniques were developed by the author.

Munyaneza was ranked first in h-index (15), despite having only 19 papers (Table 2). He contributed significantly to the prevention and control of zebra chip (ZC) caused by Candidatus Liberibacter solanacearum (CLso) carried by potato psyllid. The host preference of potato psyllid was investigated, revealing that potato psyllid infestation was influenced by host plant type, plant size, and planting density. Additionally, it was discovered that alternative hosts such as silverleaf nightshade (SLN) could sustain the population survival of psyllid and CLso. A control strategy was developed for the complete eradication of alternative hosts such as SLN from potato production fields. Tools for rapid detection of ZC were also developed based on near-infrared spectroscopy, and insecticides were utilized through Diaphorina citri picorna-like virus, present in the body of psyllid, to achieve timely detection and precise control of psyllid and CLso.

Ranked second only to the top two authors, Erik primarily delved into the analysis of plant systemic resistance mechanisms induced by disease-causing oomycetes prevalent in potatoes (Table 2). For instance, the effect of dl-aminobutyric acid treatment on potato plants was observed, directly activating defense responses by influencing hormone synthesis, amino acid metabolism, pathogenesis-related protein accumulation, and other processes. Additionally, various substances for killing disease-causing molds were developed and verified, such as biosurfactants produced by fluorescent pseudomonads, phosphite, etc., all of which contributed to the control of late blight.

BIRCH attained the highest average number of citations among the authors listed (Table 2). The primary focus of research was the investigation of the relationship between effectors produced by *Phytophthora infestans* and potato pathogen-associated molecular patterns-triggered immunity (PTI) and effector-triggered immunity (ETI). It was revealed that *Phytophthora infestans* augmented plant susceptibility to disease through the secretion of diverse effectors. Furthermore, the uptake of oomycete RxLR effectors into host cells was facilitated by clathrin-mediated endocytosis. Building upon the susceptibility mediated by effectors, nanobioprotectants were developed based on dsRNAs and phytoconjugates. These innovations provided protection by inhibiting the pathogen and enhancing the plant's defense mechanism simultaneously.

### 3.4 Author keyword analysis

**Figure 3:**
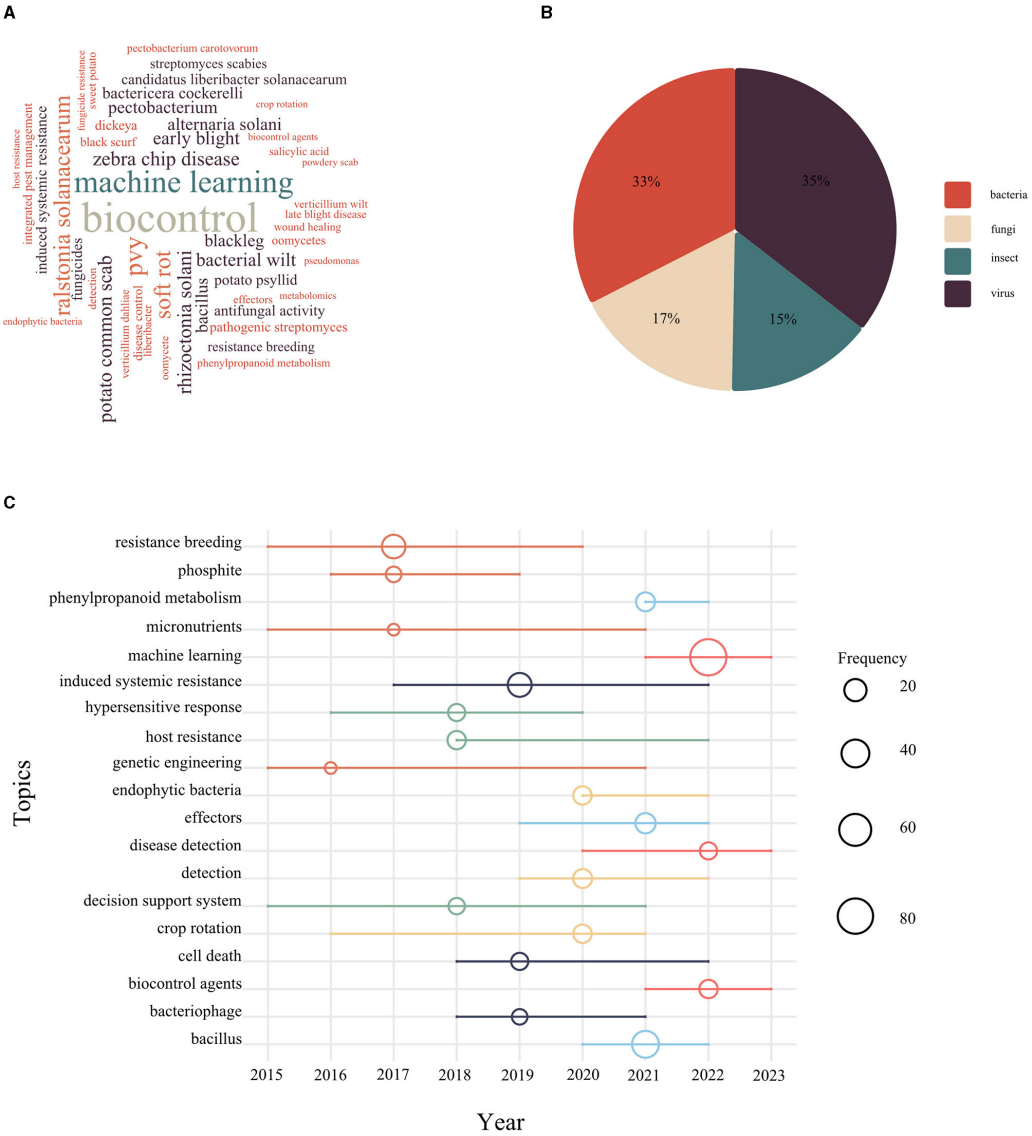
The keyword analysis of potato diseases. **(A)** Word cloud was generated based on the frequency of keywords after irrelevant words (such as disease, initial report, potato, etc.) were removed; **(B)** The proportion of disease types was calculated based on keyword statistics; **(C)** Keyword occurrences were analyzed. Circles of the same color represent the same time period, and the size of the circles represents the frequency of occurrence.

A collection of keywords based on the rich academic results of a research field over a long period of time revealed the overall characteristics, development trends, and intrinsic connections in the field of potato diseases. By de-weighting the keywords and eliminating irrelevant words, Figure 3A showed the top 50 keywords in terms of frequency, among which biocontrol, machine learning, and zebra chip disease appeared more frequently (Figure 3A). The emergent analysis diagram of prevention and control strategies based on keywords showed a shift over time. During the period from 2014 to 2017, control strategies were primarily focused on the selection of resistant varieties, including “resistance breeding,” “phosphites,” “genetic engineering,” and “micronutrients,” alongside the development of new chemical fungicides. Subsequently, from 2018 to 2020, there was a shift toward placing more emphasis on the breeding of resistant varieties and the integration of “host resistance” with tillage management practices. Between 2021 and 2023, the strategy further shifted toward utilizing beneficial microorganisms, such as *bacillus*, for “biocontrol” and adopting machine learning based technologies for rapid disease detection (Figure 3C).

### 3.5 ESI highly cited papers and co-cited network

The analysis of reference citations and co-citations provided a clear picture of the knowledge structure in the field of potato diseases and pests. **Table 4** lists the 10 most cited articles in the field of potato pests and diseases from 2014 to 2023, which could be divided into three categories according to their research content (Supplementary Figure S2). Firstly, the mechanism of the specific disease was initially analyzed, and late blight-related research constituted a significant part of the ten articles. It was found by Goss that the causal mold that triggered the Irish famine originated in central Mexico rather than in the Andes (Goss et al., 2014). Two teams, King et al. (2014) and Dagdas et al. (2016) found that effectors of *Phytophthora infestans* suppressed the potato immune system by inhibiting immune signaling and antagonizing host autophagy receptors. In addition to late blight, Charkowski discussed the changing face of bacterial soft rot, including newer detection methods and control methods. Wang et al. (2017) described the pathogenesis and control strategies for diseases caused by CLso (Charkowski, 2018). Secondly, in potato resistance breeding, the utilization of resistance gene enrichment sequencing and single-molecule real-time sequencing by Whitek et al. accelerated the cloning of potato late blight resistance genes. Schaart et al. (2016) described typical applications of various novel breeding techniques in potato (Witek et al., 2016). Finally, to assess the severity of the disease and its economic losses. Savary et al. (2019) found that national food security was related to population growth and the regional emergence of new diseases through quantitative analysis of crop losses.

The network of co-cited references can be used to analyze the relationships between the main potato diseases (Supplementary Figure S2). The purple cluster focuses on the relationship between potato psyllid and ZC, while the green cluster centers on the relationship between potato common scab and pathogenic *Streptomyces*. The blue clusters focus on the core topics of late blight and *Phytophthora infestans*, while the red clusters focus on the core topics of bacterial diseases such as blackleg and soft rot of potatoes caused by *Pectobacterium* and *Dickeya species*. We can find that research on late blight is highly popular, which could be attributed to several factors: the significant historical events caused by late blight; the more in-depth research facilitated by advances in molecular biotechnology; and the evolving causative molds, posing a new challenge for disease control (Fry et al., 2015). Compared to the other three clusters, the initial case of scab had a significantly lower number of co-citations, despite being reported over a century ago.

## 4 Discuss

### 4.1 The type of disease in the main issuing country

Potato pests and diseases were classified based on their pathogens, which included fungi, bacteria, viruses, viroid, and pests. Research findings indicated that bacterial and viral diseases received a significant share of scholarly attention, ~70%, while insect pests were the subject of fewer articles (Figure 3B). The predominant disease types in the top three countries (USA, China, and India) with the highest publication output were identified through author keywords to highlight the primary diseases under investigation (Table 3). Late blight emerged as a key focus of research in these nations due to various factors such as the pathogen's ability to evade plant defenses, its effector mechanisms that hindered host immunity and promoted susceptibility, the rapid dispersal of spores through adaptive evolution, and genetic diversity in its genome. Late blight was considered a significant threat to potato yields and quality in these regions (Leesutthiphonchai et al., 2018). In addition to late blight, fungal diseases were studied in the above countries, including black scurf, early blight, and dry rot. Bacterial diseases were the predominant focus of research in these regions, with potato common scab, bacterial wilt, and soft rot as typical diseases. Bacterial wilt was caused by *R. pseudosolanacearum*, which entered the xylem through roots or wounds and produced excessive polysaccharides that led to clogging of conduits, wilting, and ultimately death of the host plant, and the pathogen possessed a variety of alternative hosts, making it extremely difficult to control (Popa et al., 2016; Raza et al., 2016). The causal agents of soft rot, on the other hand, were *Pectobacterium atrosepticum, P. carotovorum*, and *dickeya* sp. Most of these causal agents were soil-borne and caused disease in potatoes under certain conditions of temperature and humidity (des Essarts et al., 2016).

**Table 3:** The top three countries with the highest number of publications mainly paper disease.

**USA**	**China**	**India**
Potato psyllid	Late blight	Late blight
Late blight	Potato common scab	Early blight
Zebra chip	Bacterial wilt	Tolcndv
Potato common scab	Potato dry rot	Whitefly
Soft rot	Black scurf	Black scurf
Bacterial wilt	Potato virus y	Potato virus y
Potato virus y	Early blight	Dry rot
Black scurf	Verticillium wilt	Potato common scab
Blackleg	Blackleg	
Early blight		

Tolcndv, tomato leaf curl new delhi virus.

**Table 4:** The top 10 most cited publications of ESI in potato disease field during 2014–2023.

**Paper**	**Author**	**Year**	**TC**	**Journal**	**IF (2023)**
The global burden of pathogens and pests on major food crops	Serge Savary et al.	2019	1236	Nature Ecology & Evolution	16.8
The Candidatus Liberibacter-host interface: insights into pathogenesis mechanisms and disease control	Nian Wang et al.	2017	200	Annual Review of Phytopathology	10.2
Five reasons to consider *Phytophthora infestans* a reemerging pathogen	W E Fry et al.	2015	200	Phytopathology	3.2
Threats to global food security from emerging fungal and oomycete crop pathogens	Helen N. Fones et al.	2020	167	Nature Food	23.2
Accelerated cloning of a potato late blight–resistance gene using RenSeq and SMRT sequencing	Kamil Witek et al.	2016	159	Nature Biotechnology	46.9
The changing face of bacterial soft-rot diseases	Amy O. Charkowski	2018	149	Annual Review of Phytopathology	10.2
An effector of the Irish potato famine pathogen antagonizes a host autophagy cargo receptor	Yasin F. Dagdas et al.	2016	148	eLife	7.7
Opportunities for products of new plant breeding techniques	Jan G. Schaart et al.	2016	145	Trends Plant Sci	20.5
The Irish potato famine pathogen *Phytophthora infestans* originated in central Mexico rather than the Andes	Erica M. Goss et al.	2014	145	PNAS	11.1
*Phytophthora infestans* RXLR Effector PexRD2 interacts with host MAPKKKε to suppress plant immune signaling	Stuart R. F. King et al.	2014	136	Plant Cell	12

TC, total citations; IF, impact factor.

It is important to note that in addition to potato virus y, which was being studied in various countries, tomato leaf curl new delhi virus (tolcndv) was also being researched in India, a viral disease that occurred in the tropics and subtropics and could be transmitted through insect vectors such as whitefly vectors (Pant et al., 2018). Similar to the results shown in Figure 3B, insect pests were less studied in different countries and had regional characteristics. The main insect pests of potatoes included potato psyllid, whitefly, nematodes, etc. Insects were not only mechanically damaging potato plants but also transmitting pathogens such as bacteria and viruses to plant wounds. For example, in the United States, research was mainly focused on potato psyllid, which was the vector of CLso, and ultimately triggered the ZC affecting the commercialization of potatoes (Arad et al., 2023a), whereas research on pests in India was focused on whiteflies and the spread of viruses such as tolcndv. In other words, national research on pest suppression was also based, in part, on the reduction of alternative hosts for pathogens and thus the incidence of other diseases (Kumar et al., 2021).

### 4.2 Potential disease control strategies

#### 4.2.1 Disease resistance breeding—An “opportunity” for potato breeding

Based on the highly cited analysis (Supplementary Figure S2), it can be found that breeding resistant varieties can reduce the use of pesticides while continuously resisting the invasion of pathogens, thus realizing more durable disease control and higher economic benefits. Currently, transgenic breeding, molecular marker-assisted breeding, gene silencing, and gene editing, have been applied to resistance breeding (Angmo et al., 2023; Mali et al., 2023). Hadiarto et al. incorporated the disease resistance gene RB, sourced from the wild potato species *Solanum bulbocastanum*, into the indigenous variety Granola through overexpression. Their study demonstrated that all hybrid asexual lines exhibited resistance to insects within 45–50 days post-planting (Hadiarto and Ambarwati, 2023). Of course, transgenic breeding faces challenges in its widespread adoption due to the influence of consumer populations and regional policies, hindering its rapid promotion. Traditional breeding techniques, like interspecific crosses and backcrosses, are effective but time-consuming in introducing R genes into cultivated potato varieties. In contrast, molecular marker-assisted breeding offers a solution by integrating molecular markers with specific traits to expedite the identification of target genes. Kuhl et al. (2016) had successfully created molecular markers associated with the potato leafroll virus resistance gene Rlr (etb), enabling the rapid selection of potato leafroll virus-resistant varieties through marker-assisted selection.

Gene editing technologies like the clustered regularly interspaced short palindromic repeat/CRISPR-associated protein (CRISPR/Cas) system had the potential to address public concerns regarding genetically modified (GM) products by enabling the development of GM-free alternatives. However, the efficacy of genome editing in crops utilizing the CRISPR/Cas system was contingent upon the accurate identification and targeting of specific genes (Ukhatova et al., 2023). For instance, the successful editing of the late blight susceptibility gene StERF3 using CRISPR/Cas9 technology was demonstrated by Razzaq et al. (2022), resulting in the functional suppression of the StERF3 gene and subsequently enhancing the resistance of late blight. Cas13a, a CRISPR protein that targeted RNA to provide defense against RNA phages, was utilized to cleave single-stranded RNA, resulting in the development of virus-resistant potatoes (Zhan et al., 2019). However, the challenge of polyploidy complicated genome editing in potatoes. Several optimizations for overcoming this obstacle were proposed by Mali et al. (2023), including the design of multiple gRNAs to target all alleles, targeting conserved sequences of alleles, optimizing Cas proteins, and effectively detecting off-target effects. Beyond directly editing resistance and susceptibility genes, modifying other metabolic pathways in plants could enhance disease resistance. For instance, editing the StCCoAOMT gene led to increased lignification of the potato cell wall, which restricted pathogen invasion and reduced disease severity as demonstrated by Hegde et al. (2021). Consequently, gene editing technology accelerates the breeding cycle without introducing foreign genes and stands as a key tool for developing resistant crop varieties in the future.

#### 4.2.2 Field management is an effective way to inhibit the growth and spread of pathogens

Field management encompassed the complete range of activities involved in field production, from planting to harvesting, which included practices such as tillage, irrigation, fertilization, and various other processes. Adjusting tillage methods could be utilized to decrease the presence of soil pathogens or enhance disease suppression. A notable decline in the prevalence of the pathogenic bacterium *Ralstonia solanacearum* and a significant decrease in bacterial wilt occurrences were observed following crop rotation involving wheat, maize, and potato crops by Messiha et al. (2023). Researchers also found that the incidence of potato bacterial wilt in Rwanda was affected by planting density, intercropping rotation and sharing farm tools (Uwamahoro et al., 2018). Indeed, the efficacy of agricultural practices like intercropping and crop rotation in reducing disease occurrences could be attributed to the reduction in pathogen levels by disrupting their spread and increasing the population of natural predators. Fu et al. demonstrated that intercropping onions and tomatoes could decrease the occurrence of tomato yellow wilt. Their *in vitro* experiments on the antagonistic effects of onion root secretions revealed inhibition of *Verticillium dahliae* mycelium growth and spore germination, leading to a reduction in disease incidence (Fu et al., 2015). Furthermore, Degani (2022) highlighted that practices like shallow plowing and no-tillage had been associated with decreased disease severity in specific cases. Interestingly, prolonged continuous cropping of certain crops had also been linked to a significant reduction in disease prevalence, such as the observed decline in total blight in wheat (Cook, 2003).

Water and fertilizer were deemed essential for potato growth, with appropriate irrigation and fertilization practices considered to assist in mitigating disease incidence. In a study conducted by Johansen, the relationship between soil moisture levels and potato scab occurrence was explored, revealing a notable decrease in scab incidence caused by *S. turgidiscabies* in the majority of experimental plots with increasing soil moisture content. It was suggested that irrigation could play a role in reducing scab occurrence during the early stages of potato tuber development (Johansen et al., 2015). Additionally, the impact of irrigation method choice on disease management strategies was observed by Nasr-Esfahani (2022), who noted that both sprinkler and drip irrigation methods were more effective in reducing early blight incidence caused by *Alternaria solani* compared to traditional furrow irrigation systems. Fertilizers were found to contribute essential nutrients to crops, with their application influencing soil biodiversity. Messiha et al. investigated the impact of different fertilization practices on the suppression of potato brown rot, revealing that fields with low organic matter content and carbon-to-nitrogen ratios favored the pathogen *Ralstonia solanacearum*. However, the introduction of chicken manure compost led to an increase in antagonistic bacteria like *Pseudomonas fluorescens*, resulting in enhanced disease control (Messiha et al., 2021). The utilization of organic fertilizers was shown to supply essential nutrients to the soil while simultaneously enhancing the diversity of soil microorganisms and improving the stability of the soil microbial network structure (Mawarda et al., 2020). Researchers found that the application of organic manure, such as compost, not only increased soil fertility but also decreased the abundance of *Ralstonia*, leading to a reduction in disease incidence (Messiha et al., 2023). Consequently, effective field management practices were regarded as a valuable adjunct to disease control strategies and should be integrated with other control methods to maximize efficacy and efficiency.

#### 4.2.3 Chemical control—Efficient and convenient mainstream control methods

Chemical control was the primary method utilized for disease management in agricultural practices due to its high efficacy in pest and disease eradication. Chemical pesticides, derived from natural sources like plants and microorganisms or synthetically produced, encompassed insecticides, fungicides, plant growth regulators, and other compounds (Zhan et al., 2019; Zhang et al., 2021). Notably, substances such as phosphite (Phi), essential oils, and wood vinegar solution had been extensively researched in the context of potato diseases (Figure 3C). Phi, in particular, was widely employed for controlling potato late blight, with significant inhibitory effects on oomycetes mycelial growth observed at varying concentrations (Liljeroth et al., 2020; Zhang et al., 2021; Sharma et al., 2023). Studies by Borza et al. (2019) also indicated the efficacy of Phi against potato yellow wilt, albeit requiring relatively higher concentrations for optimal results. Furthermore, aside from pesticide dosage, the method of pesticide application was crucial, and appropriate application strategies were selected based on the specific disease type. For instance, research by Hajian-Maleki et al. (2019) demonstrated that seed coating with novel essential oils resulted in a substantial reduction in the incidence of soft rot disease, while Tsror conducted a study to assess the impact of soil fumigants, specifically metam sodium (MS) and chloropicrin (PIC), on the mitigation of powdery scab in tubers. Their findings revealed a substantial reduction in both the occurrence and severity of powdery scab by 66–98% (Tsror et al., 2019). Although the application of pesticides was a prevalent method for disease management in potato cultivation, it was crucial to carefully consider the dosage and application techniques to mitigate environmental repercussions while effectively controlling diseases.

#### 4.2.4 Biological control—Environmentally friendly control methods

Biocontrol was increasingly gaining attention as a prominent area of focus in global pest management. Essentially, biocontrol aimed to suppress the proliferation of pathogens by employing mechanisms such as antagonism, predation (parasitism), resource competition, and induced plant resistance to achieve disease resistance effects (O'Brien, 2017). The soil was served as a primary reservoir for potato pests and diseases, with the inter-root zone being a battleground for the competition between beneficial bacteria and pathogens. Plant growth promoting bacteria (PGPR) represented a group of advantageous microorganisms that could be classified into two functional categories. The first category directly promoted plant growth through activities such as nitrogen fixation, phosphate solubilization, phytohormone production, and ACC deaminase production, while the second category indirectly enhanced plant growth by inducing systemic resistance, generating iron carriers, antibiotics, extracellular polysaccharides, and volatile organic acids, among other functions (Ali et al., 2022; de Andrade et al., 2023). Often, these effects occurred simultaneously. For instance, biosurfactants produced by *Pseudomonas fluorescens* were shown to rapidly degrade free-living spores within a minute, leading to a notable decrease in late blight incidence and the stimulation of protein secretion (e.g., PR proteins) in the potato variety (ovatio) following treatment with 1 mg/ml of biosurfactant (Bengtsson et al., 2015).

Several biocontrol strains had been identified as effective in disease management through various mechanisms. Research had demonstrated that *bacillus*, for instance, could trigger the expression of genes associated with the JA pathway and enhance the production of secondary metabolites in plants to bolster their defense against pest attacks (Zebelo et al., 2016). Apart from Bacillus, other commonly utilized PGPR were included *Xylaria, Streptomyces, Pseudomonas* (Mark et al., 2006; Sun et al., 2022; Shi et al., 2023). *Aspergillus* spp., a prevalent group of fungi globally, had also been investigated for its potential in disease control. For instance, researchers had isolated HNA14 from *Aspergillus* spp. and observed that the antifungal metabolites produced by this isolate significantly inhibited the growth of pathogenic *Aspergillus* spp., leading to reduced disease severity, increased plant stem height, and enhanced foliar fresh dry weight (Yao et al., 2016). *Streptomyces*, a genus of actinomycetes, comprised both pathogenic and beneficial strains. While some *Streptomyces species* could cause diseases like potato scab, others could establish symbiotic relationships with plants, offering protective benefits (Alblooshi et al., 2022). Yandigeri et al. (2015) had investigated *Streptomyces vinaceusdrappus* S5MW2 and found that its chitinase production conferred plant defense against nematode diseases triggered by *Rhizoctonia solani*. Furthermore, disrupting group sensing among pathogens could help mitigate infestations. For example, the study had discovered that *Rhodococcus pyridinivorans* XN-36 could effectively degrade N-acyl homoserine lactone produced by *Pectobacterium carotovorum*, thereby reducing the severity of soft rot diseases in carrot and potato host plants as a population burst sterilizer (Zhou Z. et al., 2022).

Biocontrol methods had been utilized to manage potato nematodes, but their effectiveness had been hindered by the nematodes' ability to create protective cysts within a gelatinous substance and survive in soil without hosts (Stirling and Stirling, 2014). The biocontrol of nematodes typically involved inducing systemic resistance in plants, leading to cellulose deposition, phenolic accumulation, or strengthening of plant cell walls through the production of biochemical metabolites (Ramamoorthy et al., 2001; Pieterse et al., 2002). Additionally, nematode populations could be reduced by introducing natural enemies of the pests (Toshova et al., 2024). Plant viruses faced challenges in infecting plants independently due to physical barriers like the cuticle and cell wall, often relying on wounds or vectors such as insects, nematodes, or fungi to reach plant cells (Hogenhout et al., 2008). The biocontrol of plant viruses primarily involved limiting pathogen spread by reducing the availability of alternative hosts (Tsror et al., 2020). *Bacillus sphaericus* stood out as an early commercially available biocontrol agent that had demonstrated effective pest control in agricultural settings against various common pests like Diptera, Coleoptera, and Lepidoptera (Wachendorff-Neumann et al., 2017; Sauka et al., 2022). While microbial preparations offered numerous advantages, caution was advised throughout their processing and application, as their efficacy in the field was influenced by factors such as microbial activity, environmental conditions, and tillage practices.

In addition to beneficial microorganisms being utilized for biocontrol purposes, ongoing development was occurring in the field of phage-based biocontrol. A novel phage, SscP1EGY, was recently identified by Abdelrhim et al., demonstrating a high lytic capacity specifically targeting *Streptomyces scabies* strains in a potato field in Egypt. The study results indicated that SscP1EGY could significantly reduce scab severity and spot area, showcasing its potential for controlling *Streptomyces scabies* infections (Abdelrhim et al., 2021). Furthermore, RNA interference (RNAi) technology emerged as a promising strategy for disease management. RNAi was typically initiated by double-stranded RNA (dsRNA), which was subsequently broken down into small interfering RNAs (siRNAs) leading to the suppression of target gene expression (Del Mar Martinez-Prada et al., 2021). For instance, a spray-based delivery method for double-stranded RNA-induced gene silencing (SIGS) to combat potato Y virus infections was developed by Samarskaya et al. (2023). Additionally, Arad et al. (2023a) successfully silenced five genes related to sugar homeostasis in the gut of the potato woodlouse using RNAi technology, resulting in over 60% mortality of the potato psyllid, suggesting a potential application of RNAi as a biopesticide target. Although biocontrol was employed in various forms, its essence was still the pursuit of friendly biological products with high control efficiency and long-lasting effects, making it one of the main control strategies for the future.

## 5 Future research directions

### 5.1 Nanoparticles—Highly efficient carriers of biologically active substances

Nanoparticles (NPs) were recognized for their advantageous characteristics such as small dimensions, extensive surface area, and numerous binding sites, making them highly effective carriers for biologically active substances like plasmid DNA and double-stranded RNA. They were increasingly acknowledged as valuable tools for enhancing the efficacy of pesticides and reducing pesticide residues (Torney et al., 2007). Various formulations, including nanoemulsions, nanosuspensions, nanogels, nanocapsules, and nanopesticides based on metal compounds, were devised to enable the slow, controlled, and targeted release of active agents (Hernandez-Tenorio et al., 2022). A self-assembled multicomponent nanobioprotectant incorporating dsRNA and phytoinducer was developed for managing potato late blight, addressing the delivery challenges of dsRNA to combat the blight fungus and extending the protective duration of RNAi (Wang Y. et al., 2023). AgNPs were synthesized by Bibi et al. (2023) utilizing various ratios of silver nitrate and Nepalese ivy extracts, resulting in complete inhibition of *Erwinia carotovora*. Nanoparticles could be loaded with traditional agrochemicals or active ingredients and combined with bioactives. A nanocapsule pesticide enhancing pest control effectiveness was described by Nuruzzaman et al. (2016), safeguarding the encapsulated active ingredient from premature degradation or prolonging its activity duration. CuO and MgO metal oxide NPs were synthesized by Rabea et al. (2023), demonstrating significant inhibition of *Ralstonia solanacearum* growth by CuO and MgO NPs at a concentration of 3 mg/mL. Consequently, NPs were seen as a promising future control strategy with broad application potential.

### 5.2 Machine learning—Accelerate the identification of targets from vast amounts of data

Machine learning and related technologies were extensively applied in the automated identification of pests and diseases affecting potato crops. A sophisticated deep learning model designed by Rashid, was introduced for recognizing diseases in potato leaves. Specifically, a convolutional neural network-based deep learning approach was devised to facilitate the training of potato plants afflicted with early and late blight. The outcomes of this study demonstrated that the model achieved an impressive accuracy rate of 99.75% when tested on a dataset of potato leaf diseases (Rashid et al., 2021). Furthermore, apart from its utilization in disease detection, scholars are employing machine learning and other technologies for disease management. A group of researchers had devised a novel machine learning methodology to pinpoint genomic markers linked to bacterial bactericidal properties. These markers were then utilized to identify additional bacteria exhibiting the desired activity, thereby enhancing the efficacy of discovering antagonistic strains (Biggs et al., 2021). Taylor utilized a blend of machine learning techniques and epidemiological methods to identify the most effective fungicide that elicits bacterial resistance at minimal levels, with the fungal pathogen *Zymoseptoria tritici* serving as a case study (Taylor and Cunniffe, 2023). Likewise, Zhang et al. integrated metadata analysis with machine learning techniques to detect both diseased and disease-suppressive soils by utilizing 54 biomarker genera. This approach aimed to elucidate overarching trends in bacterial community structure within disease-suppressive soils (Zhang et al., 2022). Therefore, leveraging machine learning methodologies can facilitate expedited identification of pertinent targets within extensive datasets, thereby expediting the processes of disease diagnosis and fungicide innovation.

### 5.3 Synthetic communities—The preventive and control effects were more stable and long-lasting

Although not included in the bibliometric analysis of this study, it was noteworthy that synthetic communities (SynComs) were considered a promising strategy for future disease management (Li et al., 2024). In contrast to conventional single-antagonist products, SynComs, comprising multiple functional microorganisms, were shown to have enhanced sustainability and efficacy in enhancing plant growth, increasing yields, and mitigating diseases (de Souza et al., 2020; Shi et al., 2023). Nevertheless, the design of SynComs was acknowledged as posing a significant challenge. For instance, a study conducted by Yin found that 10 SynComs randomly assembled from strains isolated from wheat roots did not outperform a single antagonist bacterium in controlling wheat brown rot (Yin et al., 2022). Therefore, the formulation of SynComs was emphasized to adhere to specific principles, including biodiversity, microbial interactions, stable colony structure, and effective bacteriostatic properties (Wang Z. et al., 2023). For instance, a notable reduction in Fusarium wilt disease (FWD) incidence was observed with increasing bacterial and fungal diversity. Furthermore, 10 synthetic communities based on microbial taxonomy from a pool of 205 bacterial and fungal strains isolated from the tomato root zone were constructed, revealing that cross-domain SynComs (comprising both fungal and bacterial components) were more effective in suppressing FWD compared to fungal or bacterial SynComs alone (Zhou X. et al., 2022). In general, although the current utilization of synthetic microbial communities for potato disease management was limited, their potential for application was considered substantial.

## 6 Conclusions

This study conducted a bibliometric analysis focusing on potato diseases over the past decade. The research revealed an 8.52% annual increase in publications in this field, with the United States, China, and India emerging as the primary research countries. Notably, international collaboration was particularly prevalent between China, the United States, and the United Kingdom. Variations were observed in the main diseases studied by each country, although late blight remained a common focus across all regions. The study also examined prevalent control strategies such as the utilization of resistant varieties, field management practices, chemical interventions, and biological control methods, which garnered significant attention from researchers. Through citation analysis and trending topics, potential future research directions were highlighted, including NPs, machine learning, SynComs.

## Data availability statement

The original contributions presented in the study are included in the article/Supplementary material, further inquiries can be directed to the corresponding author.

## Author contributions

LW: Writing – review & editing, Writing – original draft. ZT: Validation, Project administration, Writing – review & editing, Writing – original draft, Supervision, Methodology. MS: Writing – review & editing, Supervision, Methodology. YY: Writing – review & editing, Supervision, Data curation. KA: Writing – original draft. SL: Writing – original draft. JA: Writing – review & editing, Supervision, Methodology, Data curation. DL: Investigation, Writing – review & editing, Supervision, Methodology, Funding acquisition, Data curation.
