# Supplementary material for: Unveiling the frontiers of potato disease research through bibliometric analysis
Source: Front Microbiol. 2024 Jul 3;15:1430066. doi: 10.3389/fmicb.2024.1430066 (PMC11257026; doi:10.3389/fmicb.2024.1430066)
Supplement: Supplementary file 1 [file Data_Sheet_1.docx]

Supplementary Material

**Present situation and research trend of potato**

diseases based on bibliometrics

First Author*, Co-Author, Co-Author

*** Correspondence:** Corresponding Author: email@uni.edu

# 1 Supplementary Figures and Tables

## Supplementary Figures


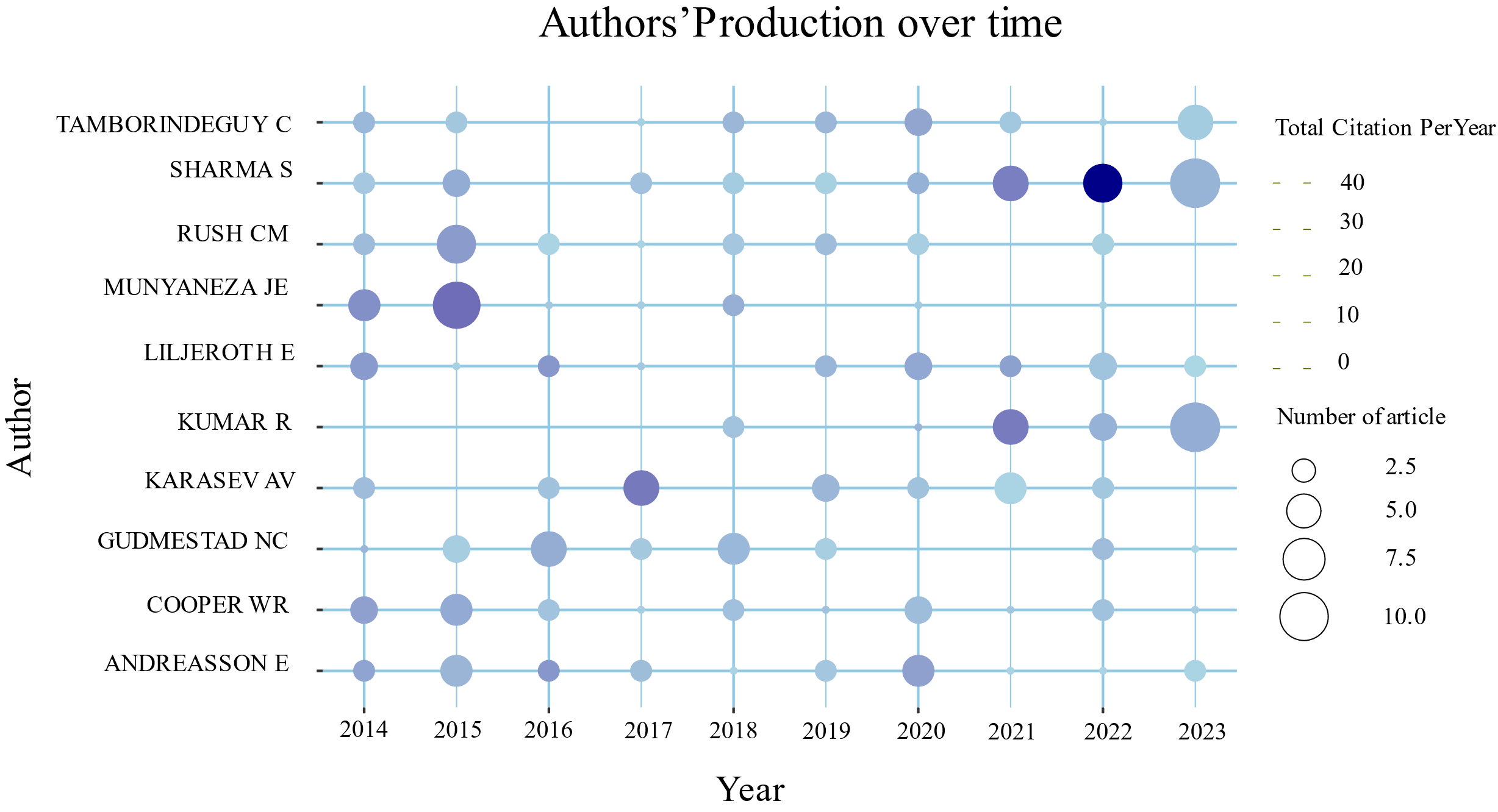


**Supplementary Figure 1.** Change in the number of articles published by authors per year. The size of the node represents the average number of citations per year, and the color of the node represents the number of posts.


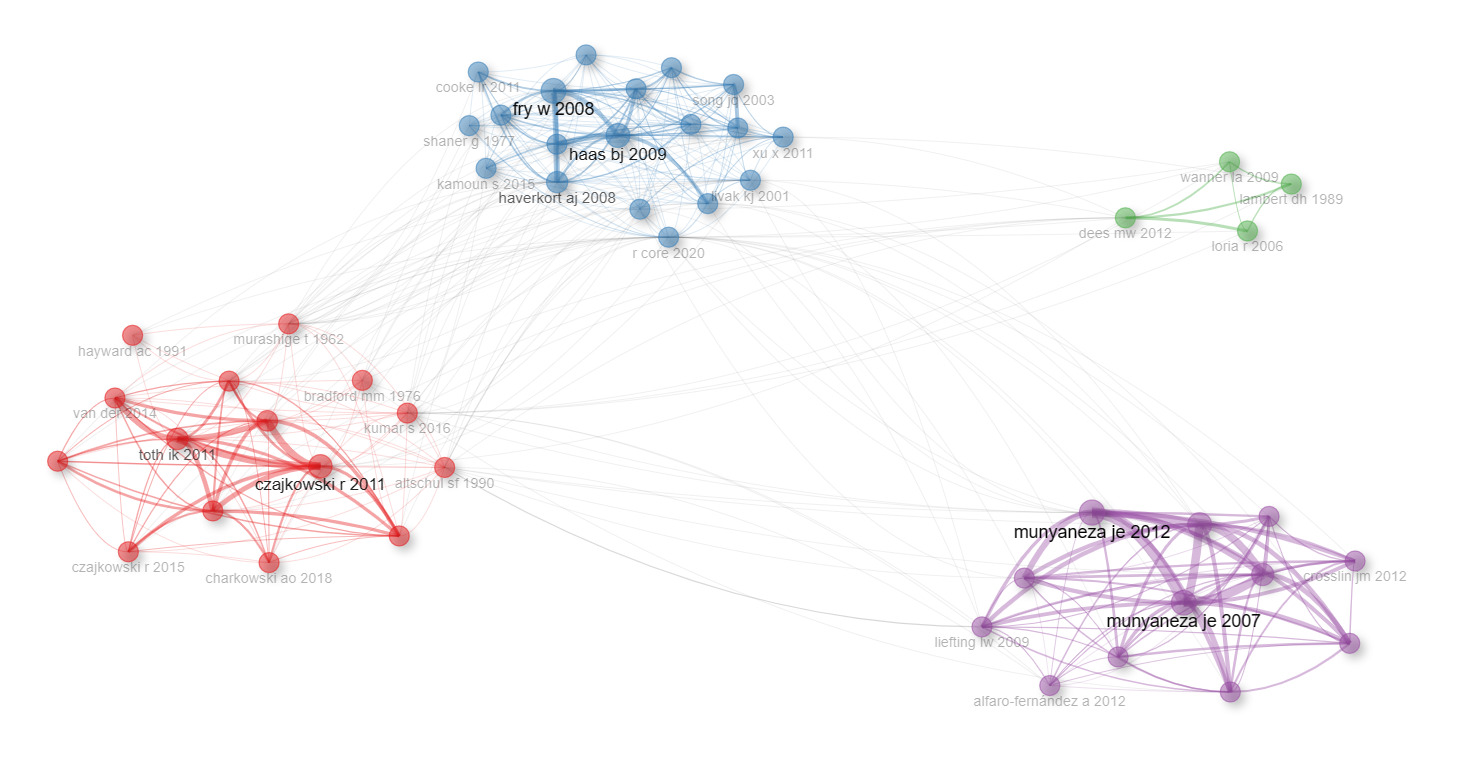


**Supplementary Figure 2.** Co-cited reference network. All co-cited articles are divided into 4 different clusters, with the size of the node representing the number of co-citations and the same color of clusters representing co-citations made during the same time period.
